# Age-related reduction of pyruvate dehydrogenase kinase 1 impairs T cell responses

**DOI:** 10.3389/fimmu.2026.1800870

**Published:** 2026-05-11

**Authors:** Rajkumar S. Kalra, Miho Tamai, Shukla Sarkar, Yong-Woon Han, Mio Miyagi, Daiki Sasaki, Hiroki Ishikawa

**Affiliations:** 1 Immune Signal Unit, Okinawa Institute of Science and Technology, Graduate University (OIST), Onna-son, Okinawa, Japan; 2 Department of Molecular Biosciences, Radiation Effects Research Foundation, Hiroshima, Japan; 3 Department of Biological Science, Faculty of Science and Engineering, Yasuda Women’s University, Hiroshima, Japan; 4 Laboratory for Integrative Genomics, RIKEN Center for Integrative Medical Sciences (IMS), Yokohama, Kanagawa, Japan; 5 Department of Molecular Oncology, Nagoya City University Graduate School of Medical Sciences, Nagoya, Japan

**Keywords:** aerobic glycolysis, aging, DIA-MS, metabolism, PDHK1, T cell activation

## Abstract

Aging is associated with impaired T cell immune responses, raising the susceptibility of the elderly to infections and cancers. Aged T cells exhibit impaired T cell responses to T cell receptor (TCR) stimulation accompanied by reduced glycolytic activity, but the molecular basis of these defects is largely elusive. Using data-independent acquisition (DIA) mass spectrometry-based proteomic analysis, we identified pyruvate dehydrogenase kinase 1 (PDHK1), a key glycolytic enzyme, as significantly downregulated in aged murine T cells. This loss of PDHK1 expression was confirmed in both CD4^+^ and CD8^+^ T cells from aged mice, regardless of their naïve or activated state. Consistent with this, aged T cells exhibited defects in the activation of glycolysis immediately after TCR stimulation. Furthermore, CRISPR-mediated *Pdhk1* deletion in young T cells led to defective activation and effector molecule expression upon TCR stimulation, whereas enforced PDHK1 expression in aged T cells facilitated these responses. These data suggest that aging diminishes PDHK1 expression in T cells, contributing to impaired glycolysis and T cell responses, which can be therapeutically restored by PDHK1 overexpression.

## Introduction

Age-related decline in T cell functions impairs cellular and humoral adaptive immune responses (1), which raises elderly people’s susceptibility to infections and cancers (2, 3). Aging compromises activation, effector, and memory functions of T cells (4, 5). These defects are attributed to an age-related reduction in naïve T cell populations (6, 7) and diminished T-cell responsiveness to antigens (8, 9); however, underlying molecular mechanisms remain largely unknown.

Cellular metabolism is central to T-cell differentiation, survival, and effector functions. Differentiation of naïve T cells to effector T cells (Teffs) depends on metabolic reprogramming from oxidative phosphorylation to aerobic glycolysis, which is required to provide energy and biosynthetic precursors for rapid T cell proliferation (10–13). Furthermore, aerobic glycolysis facilitates T cell receptor (TCR) signaling (11, 14) and supports survival and functions of T cells, as well as proliferation and migration of T regulatory cells (Tregs) (15–18). In contrast, mitochondrial oxidative metabolism, including fatty acid oxidation (FAO), is important for generation of memory T cells and for suppressive functions of T regulatory cells (Tregs) (19, 20).

In aerobic glycolysis, glycolytic end-product, pyruvate, is largely converted to lactate rather than being oxidized into acetyl-CoA in mitochondria, despite abundant oxygen availability (21). Pyruvate dehydrogenase (PDH) and pyruvate dehydrogenase kinase (PDHK) are key regulators of pyruvate fate (18). PDH catalyzes pyruvate oxidation, while PDHK inhibits PDH activity through phosphorylation, thereby promoting lactate production (22). PDHK1, one of four isoforms of PDHK family members, is activated by TCR signaling and rapidly induces aerobic glycolysis for Teffs (18, 23, 24).

Here, using data independent acquisition (DIA)-mass spectrometry (MS)-based proteomic analysis, we found a significant reduction of PDHK1 expression in aged murine T cells. Consistent with this finding, aged T cells are defective in metabolic reprogramming to glycolysis immediately after TCR stimulation. Furthermore, PDHK1 deficiency impairs TCR-induced expression of activation markers and effector molecules in young T cells, whereas PDHK1 overexpression restores expression of these molecules in aged T cells. These findings indicate that PDHK1 reduction plays a critical role in the impaired metabolic and functional responses of aged T cells.

## Results

### Age-related changes in protein expression in murine T cells

**Figure 1:**
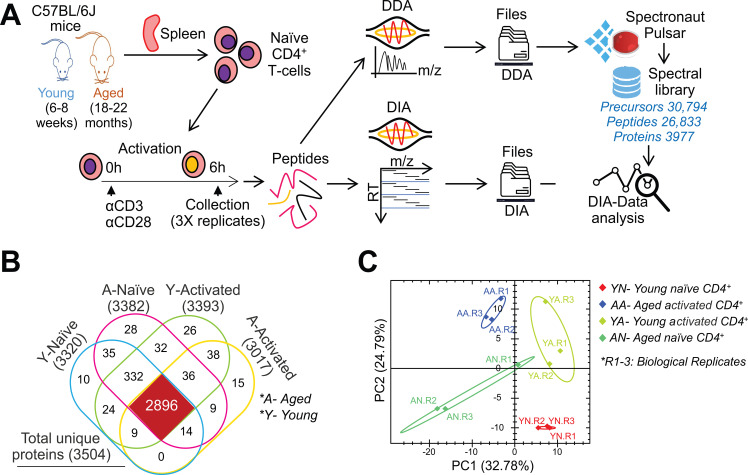
Age-related changes in protein expression in CD4^+^ T cells. **(A)** Schematic overview of sample preparation, DDA-based spectral library generation, and DIA proteomics. To prepare activated CD4^+^ T cells, naïve CD4^+^ T cells isolated from young or aged mice were stimulated with anti-CD3 and anti-CD28 antibodies for 6 h before analysis. Peptides were isolated from naive and activated CD4^+^ T cells and analyzed by DDA or DIA proteomics (n = 3 biological replicates). **(B)** Venn diagram showing the number of proteins identified by DIA proteomics in young (Y) and aged **(A)** naïve and activated CD4^+^ T cells. **(C)** PCA analysis of DIA data showing sample clustering and variance. YN, young naïve; YA, young activated; AN, aged naïve; AA, aged activated.

To investigate age-related proteomic changes in T cells, we isolated naïve CD4^+^ T cells from young (6–8 weeks) or aged mice (18–22 months), cultured either with or without activation with anti-CD3 and anti-CD28 antibodies for 6 h, and prepared samples for liquid chromatography-mass spectrometry (LC-MS) (**Figure 1A**; **Supplementary Figure S1A**). Following the construction of a spectral library of their proteome through data-dependent acquisition (DDA) in LC-MS, we performed DIA proteomic analysis of each sample group (**Figure 1A**; **Supplementary Figure S1B**). In this analysis, we detected a total of 3,504 unique proteins (**Supplementary Table S1**; **Supplementary Figures S1C,D**) with a 98.9% precursor profile recovery (**Supplementary Figures S1E–H**). Among these, 2,896 proteins were commonly detected across all sample groups (**Figure 1B**; **Supplementary Table S2**). Principal component analysis (PCA) clearly separated the four sample groups (**Figure 1C**), indicating that aging alters the proteome in both naïve and activated CD4^+^ T cells.

**Figure 2:**
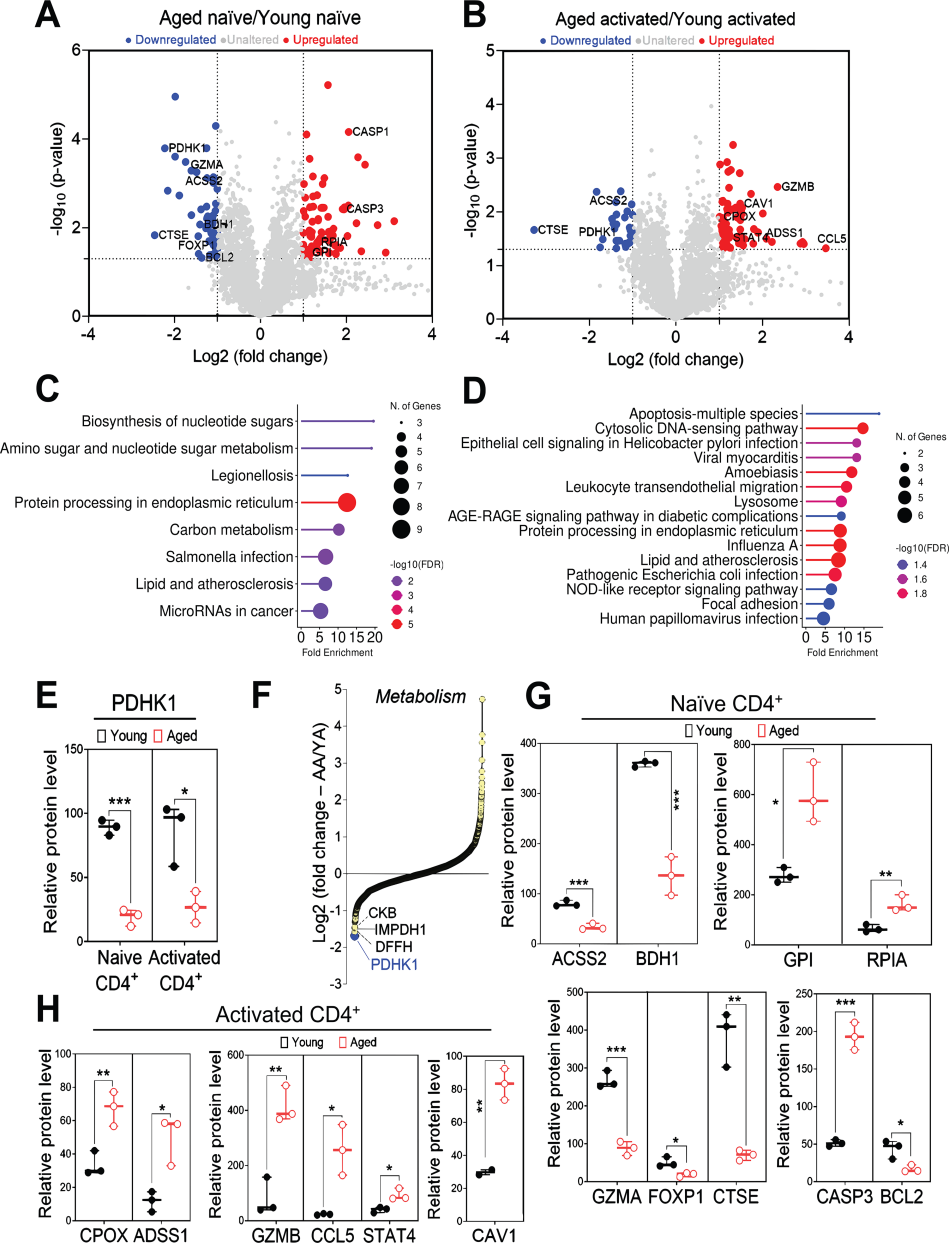
Metabolic and immune processes are altered in aged CD4^+^ T cells. Analysis of DIA data described in **Figure 1**. (**A, B)**. Volcano plot showing proteins significantly upregulated (red) or downregulated (blue) by aging in naïve **(A)** and activated **(B)** CD4^+^ T cells. The y-axis represents *p-values* (computed by 2-tailed, unequal variance (Welch’s) t-test) transformed into -log10 scale, while the X-axis shows the log2 fold change. Proteins with >2-fold change and *p* < 0.05 were considered significant. **(C, D).** GO and pathway enrichment analysis of cellular processes that significantly altered by aging in naïve **(C)** and activated **(D)** CD4^+^ T cells. -log10FDR were calculated by ShinyGO (ver.0.85) GO and pathway enrichment tool. **(E)** PDHK1 protein expression levels in young and aged CD4^+^ T cells. **(F)** Ranked list of metabolism-related proteins based on normalized protein quantity (degree of altered expression in aged cells compared to young cells). **(G, H)** Levels of key metabolic proteins and immune-related proteins in young and aged naïve **(G)** or activated **(H)** CD4^+^ T cells. **(E)**, **(G)**, **(H)** Statistical significance was assessed using a two-tailed, unpaired t-test. *p <0.05, **p < 0.01, ***p<0.001.

Using our proteome data, we sought to identify proteins whose expression is altered with aging. Analysis of naïve CD4^+^ T cells without activation showed that 44 proteins were significantly downregulated, and 61 proteins were upregulated by aging (**Figure 2A**). Additionally, analysis of activated CD4^+^ T cells showed that 56 proteins were significantly upregulated, and 28 proteins were downregulated by aging (**Figure 2B**; **Supplementary Figures S2A,B**). Gene ontology (GO) and pathway enrichment analysis revealed that GO-terms related to metabolic and immune-related processes were significantly altered by aging in naïve and activated CD4^+^ T cells, respectively (**Figures 2C,D**).

In both naïve and activated CD4^+^ T cells, we found that expression of PDHK1 (pyruvate dehydrogenase kinase 1), which promotes glycolysis (18, 23, 25), was significantly decreased by aging (**Figure 2E**). Among the metabolism-related proteins, PDHK1 showed the most pronounced down-regulation in aged naïve CD4^+^ T cells (**Figure 2F**). Additionally, aging decreased expression of a mitochondrial metabolic enzymes, BDH1 (D-beta-hydroxybutyrate dehydrogenase) and ACSS2 (acetyl-coenzyme A synthetase), while increased expression of GPI (Glucose-6-phosphate isomerase) and RPIA (Ribose-5-phosphate isomerase), which facilitate the pentose phosphate pathway (PPP), in naïve CD4^+^ T cells (**Figure 2G**). Aging also increased expression of metabolic-related CPOX (Oxygen-dependent coproporphyrinogen-III oxidase, mitochondrial) and ADSS1 (Adenylosuccinate synthetase isozyme 1) proteins in aged activated CD4^+^ T cells (**Figure 2H**). Glycolytic enzymes other than PDHK1 and LDHB (a moderate increase) were either unchanged or upregulated in the aged naive and activated CD4^+^ T cells (**Supplementary Figure S3A**). Although aged CD4^+^ T cells favor glutaminolysis (26), there was no significant age-related change in expression of glutaminolysis-related proteins (**Supplementary Figure S3B**).

We also found age-related expression changes in immune-related molecules, such as downregulation of GZMA (Granzyme A), FOXP1 (Forkhead box protein P1), and CTSE (Cathepsin E) in naïve CD4^+^ T cells (**Figure 2G**) as well as increased expression of GZMB (Granzyme B), CCL5 (CC Motif Chemokine Ligand 5), and STAT4 (Signal transducer and activator of transcription 4) in activated CD4^+^ T cells (**Figure 2H**). Other representative age-related changes included upregulation of pro-apoptotic CASP3 (Caspase-3) and downregulation of anti-apoptotic BCL2 (B-cell lymphoma 2) in naïve CD4^+^ T cells (**Figure 2G**), as well as upregulation of the endocytosis regulator CAV1 (Caveolin 1) in activated CD4^+^ T cells (**Figure 2H**). Taken together, these data suggest that aging significantly impacts protein expression related to metabolism and immune-related processes in T cells.

### Age-related decrease of PDHK1 expression in T cells

**Figure 3:**
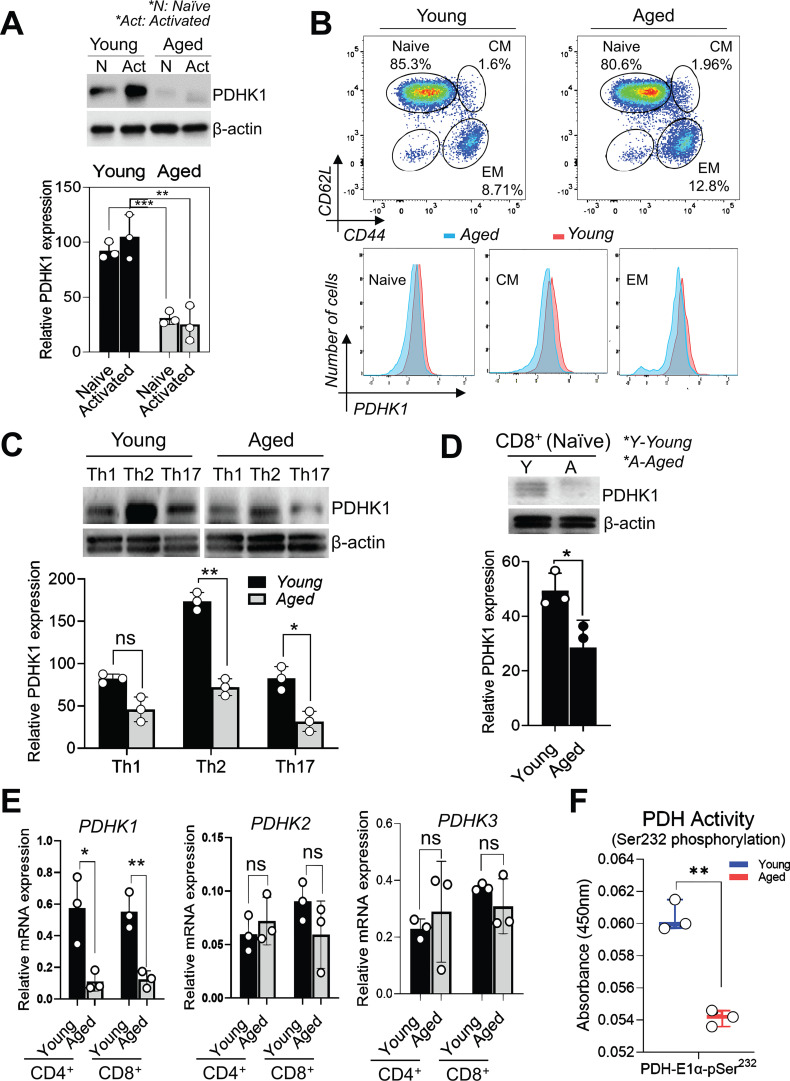
Age-related reduction in PDHK1 expression in various T cell subsets. **(A)** Immunoblot analysis of PDHK1 expression in naive CD4^+^ T cells from young and aged mice (N) and those activated with anti-CD3 and anti-CD28 antibodies for 24 h (Act). β-actin was detected as a loading control. The lower graph shows PDHK1 expression levels normalized to β-actin. **(B)** Flow cytometry analysis of PDHK1 expression in naïve (CD62L^hi^CD44^lo^), central memory (CM; CD62L^hi^CD44^hi^), and effector memory (EM; CD62L^lo^CD44^hi^) CD4^+^ T cell subsets. Upper flow cytometry plots show CD62L and CD44 expression, indicating the gating strategy for each subset, while lower histograms show PDHK1 expression in those subsets. **(C)**, **(D)** Immunoblot analysis of PDHK1 expression in young and aged T cell subsets: T helper cells (Th1, Th2, and Th17 cells) **(C)** and naïve CD8^+^ T cells **(D)**. Naïve CD4^+^ T cells were polarized to Th1, Th2, or Th17 cells for 24 h prior to analysis. PDHK1 expression levels were normalized as in **(A)**. **(E)** qPCR analysis of mRNA expression of *PDHK1*, *PDHK2*, and *PDHK3* isozymes in young and aged naïve CD4^+^ and CD8^+^ T cells. Expression levels are shown as relative values normalized to *HPRT*. **(F)** ELISA analysis of phosphorylation of PDH E1α at Ser232 in naïve CD8^+^ T cells from young and aged mice. Phospho-PDHA1 levels were normalized to total protein input. **(A–F).** The plots represent data from n = 3 biological replicates. Statistical significance was assessed using a two-tailed, unpaired t-test. ns, not significant, *p <0.05, **p < 0.01, ***p<0.001. Data are representative of 3 independent experiments.

PDHK1 facilitates pyruvate conversion to lactate in aerobic glycolysis, while inhibiting pyruvate oxidation into acetyl-CoA for mitochondrial metabolism (23, 25). Considering the essential role of glycolysis in T cell activation, we decided to focus on the age-related decrease in PDHK1 expression. We first validated the age-related reduction of PDHK1 expression in various T cell subsets. Immunoblot analysis confirmed that aged naïve and activated CD4^+^ T cells showed a significant decrease in PDHK1 expression compared to young cells (**Figure 3A**). Moreover, flow cytometry analysis of murine splenocytes revealed that aging reduced PDHK1 expression not only in naïve (CD62L^hi^CD44^lo^) CD4^+^ T cells but also in central memory (CD62L^hi^CD44^hi^) and effector memory (CD62L^lo^CD44^hi^) CD4^+^ T cell subsets (**Figure 3B**). Analysis of CD4^+^ T cells polarized into Th1, Th2, and Th17 cells also showed age-related reduction of PDHK1 (**Figure 3C**; **Supplementary Figure S4A**). Additionally, aging decreased PDHK1 expression in aged naïve CD8^+^ T cells (**Figure 3D**; **Supplementary Figure S4B**).

We next examined whether aging decreases mRNA expression of *PDHK1* and other *PDHK* isozyme genes in T cells. Quantitative RT-PCR analysis showed that *PDHK1*, *PDHK2*, and *PDHK3*, but not *PDHK4*, were expressed, and only *PDHK1* expression showed an age-related decrease in both naive CD4^+^ and CD8^+^ T cells (**Figure 3E**). Decreased PDHK1 expression implies a decline in PDH phosphorylation. To test this, we quantified phosphorylation of PDH E1α protein at serine 232 (S232) in young and aged naïve CD8^+^ T cell lysates. Phosphorylation of PDH E1α S232 is mediated by PDHK1 and leads to inactivation of the pyruvate dehydrogenase complex (29).This revealed that PDH E1α S232 phosphorylation levels were significantly lower in aged cells than in young cells (**Figure 3F**). These results indicate that aging decreases PDHK1 expression in various T cell subsets.

### Age-related impairment in TCR-induced rapid induction of aerobic glycolysis

**Figure 4:**
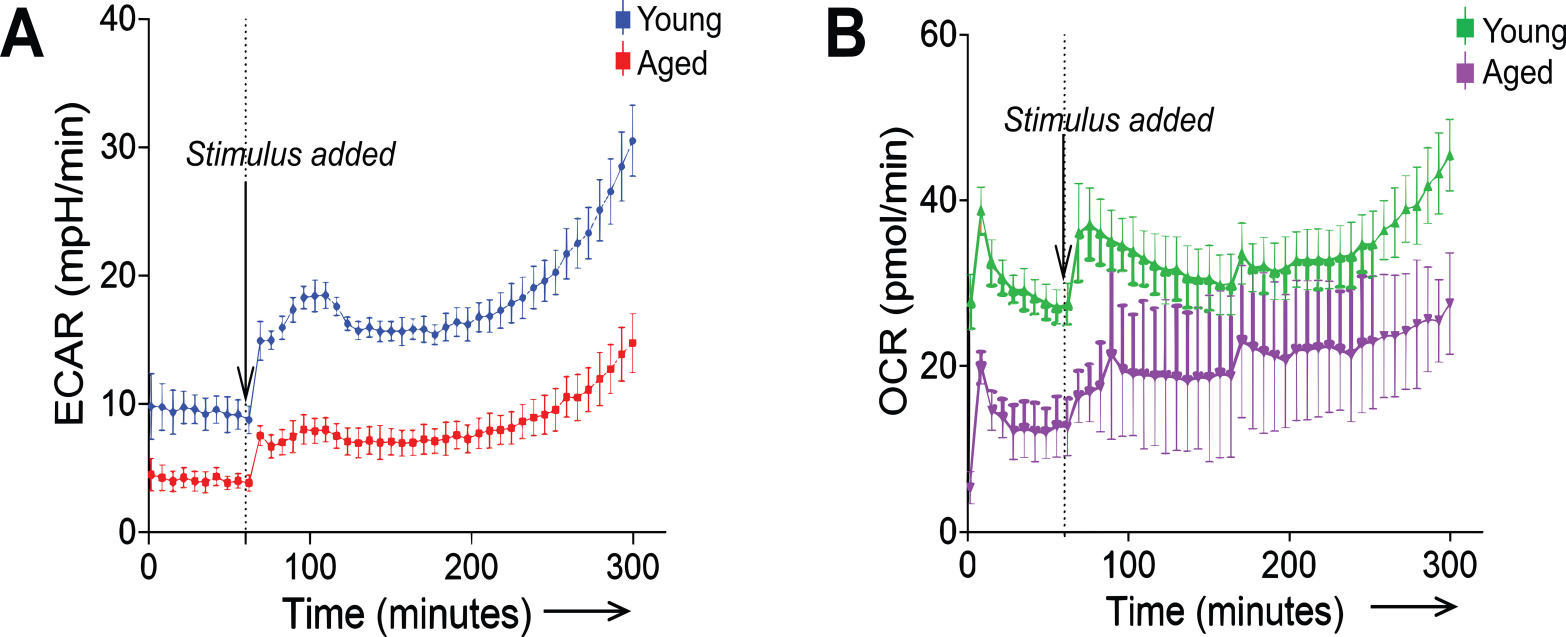
Aged naïve T cells exhibit impaired rapid induction of aerobic glycolysis. **(A, B)** ECAR **(A)** and OCR **(B)** were measured in young and aged naive CD8^+^ cells for 1 h without stimulation, followed by stimulation with dynabead-bound anti-CD3 antibody and anti-CD28 antibody for 4 h. **(A, B)** Error bars represent standard deviations (SD). The plots represent data from n = 3 biological replicates.

The steady-state PDHK1 expression allows naïve T cells to rapidly induce aerobic glycolysis within a few hours after TCR stimulation, independently of upregulation of glycolytic gene expression, as shown in CD8^+^T cells (23). Accordingly, our finding of the age-related decrease in PDHK1 expression in naïve T cells suggests that aged T cells are defective for TCR-induced rapid induction of glycolysis. However, although previous studies reported that aged CD4^+^ and CD8^+^ T cells exhibit defective glycolysis in the late phase after TCR stimulation (5, 27, 28), it remains unclear whether they have defects in the induction of glycolysis immediately after TCR stimulation. To address this, we activated CD8^+^ T cells with TCR stimulation and analyzed the kinetics of their extracellular acidification rate (ECAR) and oxygen consumption rate (OCR), which are indicators of glycolysis and oxidative phosphorylation, respectively. Because the role of PDHK1 in the early induction of glycolysis has been well established in CD8^+^ T cells (23), we examined age-associated defects in early glycolytic activation in CD8^+^ T cells. This revealed that both ECAR and OCR levels, before and within 4 h after TCR stimulation, were significantly lower in aged CD8^+^ T cells compared to young CD8^+^ T cells (**Figures 4A,B**). These results suggest that the rapid induction of glycolysis following TCR stimulation is impaired in aged T cells, likely due to an age-related decrease in PDHK1 expression.

### PDHK1 promotes T cell activation and functions

Aerobic glycolysis is not only essential for energy generation in massively proliferating T cells but also promotes various T cell responses. For example, using RNAi knockdown or dichloroacetate (DCA), an inhibitor of PDHKs, previous studies suggested that PDHK1 deficiency impairs Th17 differentiation of CD4^+^ T cells (18) and inflammatory cytokine expression in CD8^+^ T cells (23). To further explore the impact of PDHK1 deficiency in T cells, we next assessed the impact of CRISPR/Cas9-mediated knockout of the *PDHK1* gene on CD4^+^ T cell activation.

**Figure 5:**
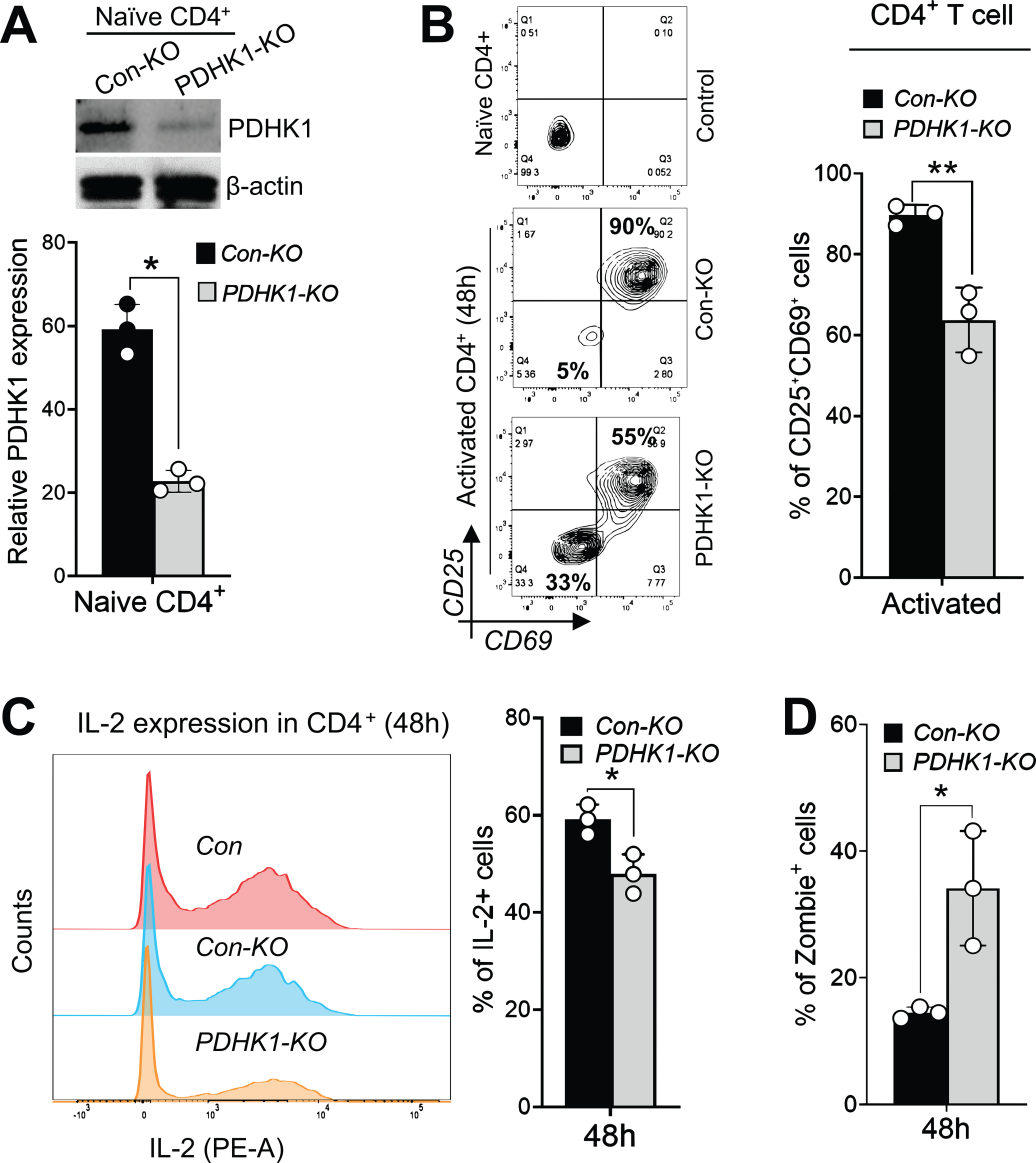
PDHK1 knockdown compromises immune function in young CD4^+^ T cells. **(A)** Immunoblot analysis of PDHK1 expression in naïve CD4^+^ T cells transduced with either control CRISPR-RNP (con-KO) or PDHK1-targeting CRISPR-RNP (PDHK1-KO). Cells were cultured for 48 h after electroporation prior to analysis. β-actin was detected as a loading control. The lower graph shows PDHK1 expression levels normalized to β-actin. **(B–D)** Flow cytometry analysis of CD25 and CD69 expression **(B)**, IL-2 expression **(C)**, or frequency of dead cells **(D)** in con-KO or PDHK1-KO CD4^+^ T cells upon TCR-stimulation. Naïve con-KO and PDHK1-KO cells were cultured for 48 h after CRISPR-RNP electroporation, then stimulated with anti-CD3 and anti-CD28 antibodies for an additional 48 h. CD25 and CD69 expression was then analyzed, while IL-2 expression was analyzed after restimulation with PMA/ionomycin for 4 h. Dead cells were detected by staining with zombie dye at 48 h after stimulation of naïve cells **(D)**. **(A–D).** The plots represent data from n = 3 biological replicates. Statistical significance was assessed using a two-tailed, unpaired t-test. ns, not significant, *p <0.05, **p < 0.01.

Immunoblot analysis confirmed a significant reduction in PDHK1 expression in naïve CD4^+^ T cells after electroporation with PDHK1-specific CRISPR RNA (crRNA) and CAS9 protein (**Figure 5A**). Flow cytometry analysis after TCR stimulation revealed that PDHK1 deficiency significantly reduced the frequency of cells expressing activation markers, CD69 and CD25 (**Figure 5B**). Reduced expression of CD69 and CD25 was also observed in young CD4^+^ T cells treated with the PDHK inhibitor DCA (**Supplementary Figure S5A**).

PDHK1 deficiency also decreased restimulation-induced expression of IL-2 in activated CD4^+^ T cells (**Figure 5C**). Additionally, young CD4^+^ T cells with PDHK1 deficiency exhibited increased cell death (**Figure 5D**; **Supplementary Figure S5B**). In contrast, analyses of CFSE dilution showed that PDHK1 deficiency did not influence proliferation of activated CD4^+^ T cells up to 96 h (**Supplementary Figure S5C**). These results indicate that PDHK1 is indispensable for activation and survival of naïve CD4^+^ T cells upon TCR stimulation, but it is not strictly required for their proliferation.

### PDHK1 overexpression improves activation, survival, and effector function in aged T cells

**Figure 6:**
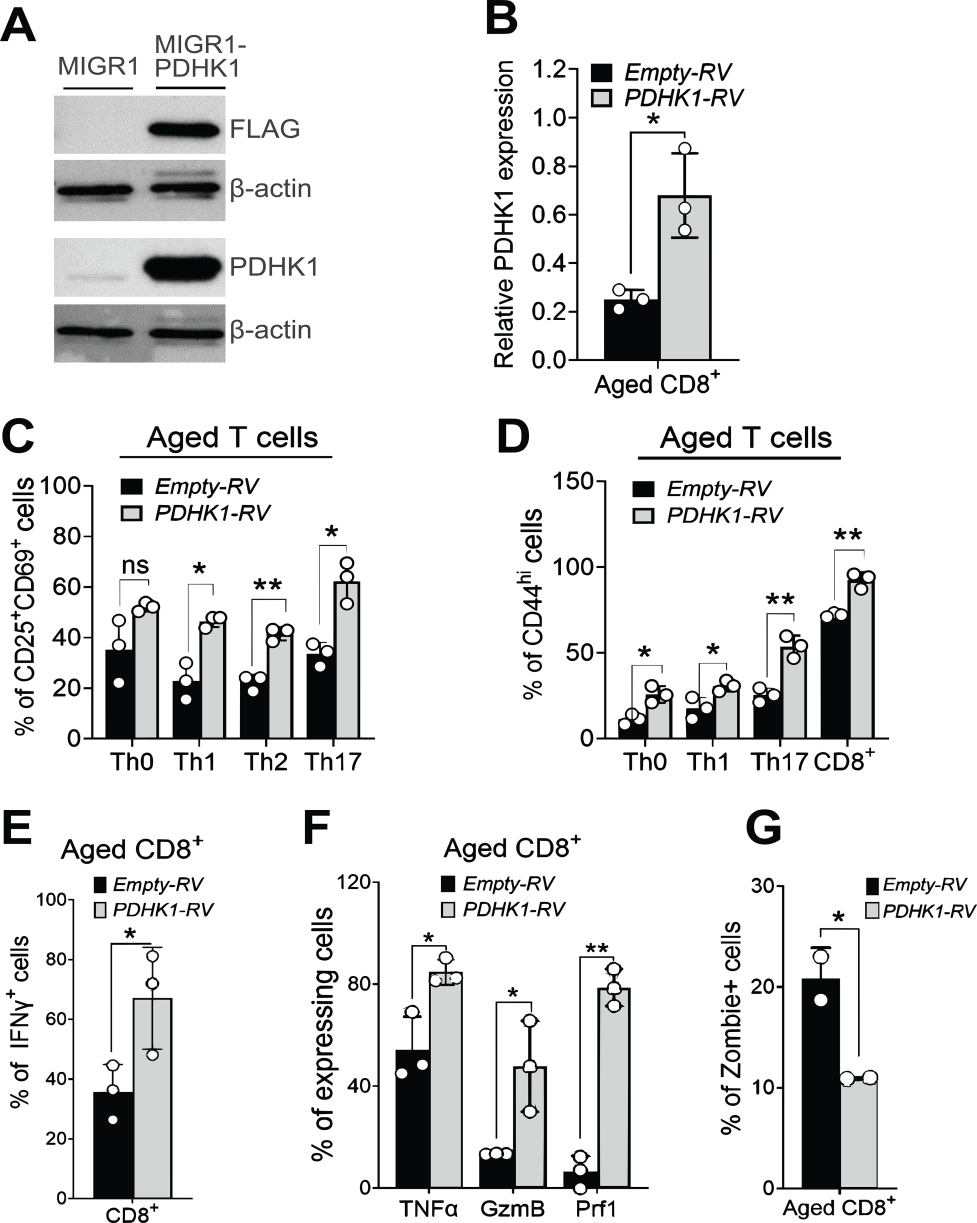
PDHK1 overexpression improves activation, survival, and effector molecule expression in aged T cells. **(A)** Immunoblot analysis of exogenous PDHK1 expression in Plat-E cells at 48 h post-transfection with MIGR1 empty vector or MIGR1 vector encoding Flag-tagged PDHK1. Antibodies against Flag, PDHK1, and β-actin were used. **(B)** qPCR analysis of *PDHK1* mRNA expression in aged CD8^+^ T cells transduced with either control retrovirus (Empty-RV) or Flag-PDHK1–expressing retrovirus (PDHK1-RV). Cells were analyzed at 48 h post retroviral infection. Expression levels are shown as relative values normalized to *HPRT*. **(C)** Flow cytometry analysis of CD25 and CD69 expression in T helper cells transduced with Flag-PDHK1. CD4^+^ T cells transduced with retroviral vectors were cultured for 48 h after infection, followed by polarization to T helper subsets for 48 h prior to analysis. **(D)** Flow cytometry analysis of CD44 expression in T helper and CD8^+^ T cells transduced with Flag-PDHK1. Cells were transduced and activated as described in **(C)**. **(E–G)** Flow cytometry analysis of IFN-γ expression **(E)**, TNFα, GzmB, and Prf1 expression **(F)**, and frequency of dead cells **(G)** in CD8^+^ T cells transduced with Flag-PDHK1. CD8^+^ T cells transduced with retroviral vectors were cultured for 48 h after stimulation with anti-CD3 and anti-CD28 antibodies. 48 h later, cells were restimulated with PMA/ionomycin for 4 h, and IFN-γ TNFα, GzmB, and Prf1 expression was analyzed **(E, F)**. **(A–E)** n = 3 biological replicates were analyzed. Statistical significance was assessed using a two-tailed, unpaired t-test. ns, not significant, *p <0.05, **p < 0.01. Data are representative of 2 or 3 independent experiments.

Finally, to test whether PDHK1 overexpression can improve the activity of aged T cells, we retrovirally transduced the *PDHK1* gene into TCR-stimulated naïve T cells isolated from aged mice. After confirming exogenous PDHK1 expression (**Figure 6A**) and increased *PDHK1* mRNA in transduced CD8^+^ T cells (**Figure 6B**), we examined the effect of PDHK1 overexpression in aged CD4^+^ or CD8^+^ T cells on expression of key genes induced by TCR stimulation. We found that overexpression of PDHK1 significantly increased expression of activation markers, CD25, CD69, and CD44 in activated CD8^+^ T cells as well as CD4^+^ T cells activated under polarizing conditions for Th1, Th2, and Th17 cells (**Figures 6C,D**; **Supplementary Figures S6A,B**). Further analyses of activated CD8^+^ T cells revealed that PDHK1 overexpression enhanced expression of effector molecules, such as IFN-γ, TNF-α, GzmB, and perforin, as well as the cell viability (**Figures 6D–G**; **Supplementary Figures S6C–E**). These results demonstrate that PDHK1 overexpression in aged T cells can improve their activation, survival, and effector functions.

## Discussion

In this study, using a DIA-MS-based proteomics, we identified PDHK1, the key kinase to promote glycolysis, as a protein significantly downregulated both in naïve and activated CD4^+^ T cells. We confirmed that the age-related decrease in PDHK1 expression is consistent in both CD4^+^ and CD8^+^ T cells. We also observed that aged CD8^+^ T cells are defective in TCR-induced rapid induction of aerobic glycolysis, which is dependent on PDHK1 (23). Analysis of young naïve T cells with CRISPR-mediated PDHK1 deficiency revealed PDHK1’s roles in enhancing expression of T cell activation markers (CD25 and CD69) and IL-2, as well as supporting cell survival. Notably, we demonstrated that retroviral overexpression of PDHK1 increases expression of T-cell activation markers and effector molecules in aged CD4^+^ and CD8^+^ T cells upon TCR stimulation and promotes their survival. These results suggest that age-related reduction of PDHK1 expression in T cells impairs not only aerobic glycolysis but also T-cell responses to antigens.

PDHK1 deficiency and aging have similar effects on various T cell responses. TCR-stimulated aged mouse CD4^+^ T cells exhibit lower levels of cell proliferation, viability, expression of activation markers and IL-2, and aerobic glycolysis, compared to young cells (1, 8, 9, 30). We observed that PDHK1 deficiency causes similar defects in these responses, except for cell proliferation, in young CD4^+^ T cells. Consistently, Menk et al. observed that inhibition of PDHK activity by DCA in young CD8^+^ T cells significantly decreased expression of inflammatory cytokines, including IL-2, but had only a minor effect on cell proliferation (23). Thus, the age-related decrease in PDHK1 expression appears to be closely associated with various defects in T cell activity.

Whether and how PDHK1 overexpression improves metabolic defects in aged T cells remains unclear and should be addressed in future studies. As discussed in a previous report (23), T cells with PDHK1 deficiency appear to support metabolic needs for TCR-induced cell proliferation by changing pyruvate flow into mitochondrial oxidative phosphorylation. However, because both glycolytic and mitochondrial metabolic pathways are defective in aged T cells (5, 27), which is supported by our ECAR and OCR data (**Figure 4**), decreased expression of PDHK1 may underlie metabolic failure in aged T cells. Although PDHK1 overexpression is expected to promote aerobic glycolysis, it is unlikely to restore the defects in mitochondrial metabolism in aged T cells. In this context, a thorough evaluation of how PDHK1 overexpression affects metabolic pathways in aged T cells is warranted. Furthermore, because our current analysis focuses only on early T-cell activation, future studies should also determine its impact on broader aspects of T-cell biology linked to cellular metabolism, including proliferation and long-term maintenance of activated T cells.

Multiple mechanisms may be involved in how PDHK1 deficiency affects the expression of genes associated with T cell activation. PDHK1 inhibits PDH-mediated conversion of pyruvate to acetyl-CoA for mitochondrial metabolism, thereby promoting lactate dehydrogenase (LDH)-mediated conversion of pyruvate to lactate in aerobic glycolysis (23, 31). In CD8^+^ T cells, it has been demonstrated that PDHK1 deficiency frees LDH from its role in aerobic glycolysis and allows LDH to suppress translation of mRNA of inflammatory cytokine genes such as *Il2*, *Tnfa*, and *Ifng*, by binding to AU-rich motifs (23). This mechanism may also contribute to CD4^+^ T cells, as we observed reduced IL-2 levels in PDHK1-deficient CD4^+^ T cells. The effect of PDHK1 deficiency on the expression of other genes induced by TCR stimulation may be due to impairments of several pathways dependent on glycolysis. This is supported by the notion that glycolysis facilitates mTOR, glutaminolysis, and TCR signaling pathways, thereby enhancing expression of some molecules, including CD25, during T cell activation (32). Interestingly, although it has been shown that PDHK1 deficiency did not affect expression of cytolytic molecules, perforin, and GZMB in CD8^+^ T cells (23), we found that overexpression of PDHK1 increases expression of these cytolytic molecules in aged CD8^+^ T cells. This may reflect a differential dependence on PDHK1 for activation between young and aged T cells.

In conclusion, our findings suggest that aging decreases PDHK1 expression, thereby impairing various T cell responses induced by TCR stimulation. Moreover, we demonstrated that PDHK1 overexpression can enhance the activity of aged T cells *in vitro*. Although further *in vivo* studies are needed to establish translational relevance, our results highlight PDHK1 as a promising target for restoring T-cell immunity in the elderly.

## Methods

### Mice

C57BL/6 mice were obtained from Clea (Tokyo, Japan). All mice were maintained in specific pathogen-free conditions. Gender-matched 6–8-week-old (young) and 18–22-month-old (aged) mice were used for experiments. All mouse experimental protocols were approved by the institutional animal care and use committee at the Okinawa Institute of Science and Technology (OIST) Graduate University.

### Reagents and antibodies

Reagents utilized in proteomics included ammonium bicarbonate (09830, Fluka), trypsin/Lys-C Mix (V5073, Promega), iodoacetamide (IAA) (095-02151, Wako), dithiothreitol (DTT) (041-08976, Wako), acetic acid (695092, Sigma), acetonitrile (018-19853, Wako), methanol (#106018; Merck), formic acid (#A117-1AMP; Fisher), tube-adaptor 10-200 μL tip (5010-21514, GL Sciences), MonoSpin C18 columns (#5010-21670, GL Sciences), Dynabeads protein G (#10004D; Thermo), sodium pyruvate (#P2256; Sigma-Aldrich). Following antibodies were used for flow cytometry analysis at a 1:200 dilution: anti-CD3 (17A2; Biolegend), anti-CD4 (GK1.5; Biolegend), anti-IL-4 (11B11; Biolegend), anti-CD28 (37.51; Biolegend), anti-CD25 (PC61; Biolegend), anti-CD69 (H1.2F3; Biolegend), anti-CD44 (IM7; Biolegend), anti-CD62L (MEL-14; Biolegend), anti-IL-2 (JES6-5H4; Biolegend), anti-IFNγ (XMG1.2; Biolegend), anti-TNFα (MP6-XT22; Biolegend), anti-Granzyme B (GB11; Biolegend), anti-Perforin (S16009A; Biolegend), and anti-rabbit IgG (Poly4064; Biolegend). Following cytokines were used: IL-2 (#575404; Biolegend), IL-12 (#577002; Biolegend), IL-4 (#574306; Biolegend), IL-6 (#575706; Biolegend), TGF-β1 (#100-21C; PeproTech), IL-15 (#566302; Biolegend). For immunoblotting and immunofluorescence, the following antibodies were used: anti-PDHK1 (C47H1; Cell Signaling), anti-FLAG (#F1804; Sigma-Aldrich), and anti-β-Actin (AC-15; Sigma-Aldrich), IgG-HRP (#7076S; Cell Signaling), and anti-rabbit IgG-HRP (#7074S; Cell Signaling).

### T cell isolation and activation

Naïve CD4^+^ T cells were collected from murine spleens and purified using MojoSort mouse naïve CD4^+^ selection kits (#480040; Biolegend, USA) according to the manufacturer’s instructions. For naïve CD8^+^ T cell purification, the MojoSort mouse naïve CD8 T cell isolation kit (#480044; Biolegend) was used. For total CD4^+^ and CD8^+^ T cell purification, MojoSort CD4^+^ and CD8^+^ specific selection kits (#480033 and #480035, respectively) were used. The purity of cells (93-97%) was confirmed by FACS Fortessa (BD). Purified naïve and total CD4^+^ T cells were cultured in IMDM medium (Invitrogen) supplemented with 10% fetal bovine serum (FBS; Sigma-Aldrich), 1X streptomycin-penicillin (Sigma-Aldrich), and β-mercaptoethanol (55 μM; Invitrogen). For T cell activation, complete IMDM medium was further supplemented with anti-CD28 antibody (1 μg/mL) in either 24-well or 48-well plates (seeded at 2x10^5^ or 1x10^5^ cells/well, respectively) pre-coated with anti-CD3 antibody (5 μg/mL).

For Th cell polarization, IMDM medium was further supplemented with IL-2 (20ng/mL), anti-IFNγ (1 μg/mL), and anti-IL-4 (1 μg/mL) for Th0; IL-2 (20ng/mL), IL-12 (20 ng/mL), and anti-IL-4 (1 μg/mL) for Th1; IL-2 (20 ng/mL), IL-4 (100 ng/mL), and anti-IFNγ (1 μg/mL) for Th2; IL-6 (20 ng/mL) and TGF-β1 (3 ng/mL) for IL-23-independent non-pathogenic Th17 differentiation. Naïve and total CD8^+^ T cells were cultured in PRMI-1640 (Nacalai Tesque) medium supplemented with 10% FBS, 1X streptomycin-penicillin, 55 μM β-mercaptoethanol, 10 nm non-essential amino acids (NEAA; Gibco), sodium pyruvate, and 1mM HEPES. For CD8^+^ T cell activation, complete RPMI-1640 medium was supplemented with α-CD28 antibody (1 μg/mL) in 48-well plates pre-coated with anti-CD3 antibody (5 μg/mL). Cells were harvested for assays at the time points indicated in the figure legends.

### Flow cytometry

To analyze cell surface markers, cells were stained with antibodies and Zombie-NIR (423106; Biolegend;1:400 dilution) in phosphate-buffered saline (PBS) containing 2% FBS for 30 mins on ice, followed by two washes. To analyze intracellular markers, cells were fixed, permeabilized, and stained with a Foxp3 Staining Buffer Set (00-5253-00; eBioscience), following the manufacturer’s instructions. For intracellular cytokine expression analysis, T cells were restimulated with phorbol 12-myristate 13-acetate (PMA; 50 ng/mL; Sigma) and ionomycin (500 ng/mL, Sigma) in the presence of brefeldin-A (5 mg/mL; Biolegend) for 4 h. T cells were then fixed with 4% paraformaldehyde (PFA), permeabilized with permeabilization/wash buffer (Biolegend), and stained with specific antibodies.

For cell proliferation analysis, T cells were labeled with 2 μL of carboxyfluorescein succinimidyl ester (CFSE; 423801; Biolegend) in 1 mL of 1X PBS for 20 min at ice and washed with PBS containing 2% FBS. Cells were subsequently cultured under conditions specified in each figure legend and analyzed by flow cytometry. For cell count and viability analyses, cells (in 4 μL suspension) were collected in a tube at different time points, diluted with PBS (56 μL), and mixed with 60 μL (30X dilution) Muse™ Count & Viability Reagent (MCH100102, Millipore) and incubated for 5 mins in the dark. Samples were then analyzed using Muse™ Cell Analyzer (Millipore).

Flow cytometry data were analyzed with FlowJo (FlowJo LLC, USA) software. Flow cytometry gating was based on unstained and fluorescence-minus-one (FMO) controls, as well as unstimulated samples with corresponding FMOs. Gates were applied uniformly across samples.

### Proteomics sample preparation

Naïve or 6-h activated CD4^+^ T cells were first lysed in SDT buffer (4% SDS, 100 mM DTT, 100 mM Tris-HCL, pH 7.6) and sonicated (70 amplitude) in a QSONICA sonicator (Q800R) for 30 sec ON and OFF cycles, repeated ten times at 4 °C using a chiller. Samples were then precipitated with acetone at -20 °C for 1 h, washed twice with acetone by centrifugation at 15,000 x g for 5 min at 4 °C, air-dried for 10 min, and resuspended in 40 μL of the sample buffer (100 mM Tris-HCl, pH 9, 2 mM sodium deoxycholate [SDC), 12 mM sodium N-lauroylsarcosinate [SLC]). Samples were briefly vortexed and sonicated for 15 min, and their concentration was estimated using Pierce BCA protein assay kit (#23225; Thermo). Samples were then reduced with 100 mM DTT (50 °C for 30 mins), alkylated with 200 mM IAA for 30 min at room temperature [RT] in the dark, and 400 mM Cysteine for 10 min at RT. Samples were then diluted with 160 μL ammonium bicarbonate (ABC) and digested with Lys-C (200 ng/μL) and trypsin (100 ng/μL) at 37 °C overnight. Peptides were then cleared for SDC and SLC by mixing with ethyl acetate and 5% trifluoroacetic acid (TFA), followed by centrifugation at 12,000 x g for 5 min at 25 °C and freezing for 15 min at -80 °C, and removing the upper liquid layer. Peptides were then dried using a SpeedVac concentrator for 1 h to remove ethyl acetate and diluted with 100 μL of 0.1% TFA. Desalting and purification of peptides were performed with MonoSpin C18 columns (#5010-21670, GL Sciences). The column was first equilibrated by sequential washes with methanol, 80% acetonitrile (ACN) containing 0.1% TFA, and 3% ACN containing 0.1% TFA (200 μL each) by centrifugation at 2,300 x g for 1 min at RT. 100 μL of peptide solution was then loaded onto the column, washed twice with 3% ACN containing 0.1% TFA, and eluted in a fresh sterile tube with 50% ACN containing 0.1% TFA. Purified peptides were dried using a SpeedVac and redissolved in 20 μL of 3% ACN containing 0.1% TFA. Prior to LC-MS/MS injections, peptide quantities were estimated using a BCA assay.

### DIA-MS

A preliminary data-dependent acquisition (DDA) was performed for SWATH protein quantification, as described previously (33). Peptides (approx. 200 ng) were directly injected onto a 100 μm × 15 cm PicoFrit emitter (New Objective) packed in-house with 120-Å porous C18 particles (ReproSil-Pur C18-AQ 1.9 μm; Dr. Maisch GmbH; pC18_B) and then separated by using a 240-min acetonitrile gradient (3%–40%; flow rate, 300 nL/min) and an Eksigent Ekspert nanoLC 400 HPLC system (Sciex). Peptides eluting from the column were analyzed on a TripleTOF 5600+ mass spectrometer (Sciex). MS1 spectra were collected in the range of 400–1200 *m*/*z* for 250 ms. The top 25 precursor ions with charge states of 2+ to 5+ that exceeded 150 counts per second were selected for fragmentation with rolling collision energy, and MS2 spectra were collected for 100 ms. A spray voltage of 2100 V was applied. SWATH data-independent acquisition (DIA) was performed by using the same gradient profile as used for the data-dependent acquisition experiments described above. Precursor ion selection was done in the 400–1200 *m*/*z* range, with a variable window width strategy (7–75 Da). The collision energy for each individual SWATH experiment was set at 45 eV, and 80 consecutive SWATH experiments (100–1800 *m*/*z*) were performed, each lasting 36 ms. Data-independent acquisition raw data were analyzed by using the SWATH processing function embedded in the PeakView software (Sciex). Peptides from pig trypsin and protease I precursor lysyl endopeptidase were used for retention time recalibration between runs. The following criteria were used for data-independent acquisition quantification: peptide confidence threshold, 99%; 30 ppm maximum mass tolerance; 6 min maximum RT tolerance. Multivariate data analysis was performed by using Markerview software (Sciex). MS data in triplicated samples was acquired in DDA and DIA modes.

The triplicated DDA files from sample sets were taken for spectral library generation by using the Pulsar algorithm integrated into Spectronaut 18.5 software (Biognosys) against the mouse UniProt FASTA file database (version 2024_03). In the search settings, enzyme specificity was set to trypsin/P, allowing two missed cleavages. Fixed modifications were set to Carbamidomethyl (C), and variable modifications included oxidation (M) and acetylation (protein N-terminus). False discovery rate (FDR) values for the protein, peptide, and peptide-spectrum match (PSM) were set to 0.01. For MS1 and MS2 tolerances, dynamic settings were used with a correction factor of 1. The rest of the parameters were set as default. The generated spectral library from DDA samples was used for quantitative data analyses in the Spectronaut 18.5 software suite. The cutoffs for the precursor q-value and experiment protein q-value were set to 0.01. The remaining parameters were set as default. Results from sparse profiles included peptides identified with q-value < 0.01 in at least one sample. The software’s graphical interface was used to retrieve matrix and quantitative readouts, as shown in **Supplementary Figure S1**. Gene ontologies and gene-set enrichment analysis were performed with ShinyGO and DAVID.

### Immunoblotting

Immunoblotting was performed as described previously (34). Briefly, proteins were extracted in RIPA buffer (Wako) with a protease inhibitor cocktail (Roche). Cleared lysates were denatured with 5x loading buffer (250 mM Tris-HCl, pH 6.8, 5% β-mercaptoethanol, 30% glycerol, 10% SDS, and 0.1% bromophenol blue) and resolved by SDS polyacrylamide gel electrophoresis (SDS-PAGE). Proteins were transferred from gels to Immobilon-P membranes (Millipore) using a wet transfer system (Bio-Rad). In parallel, separate gels were stained with Coomassie Brilliant blue (#1610436; Bio-Rad). Membranes were blocked with 5% bovine albumin (Wako) in Tris-buffered saline with 0.1% Tween-20. Membranes were then incubated with indicated antibodies (diluted according to the manufacturer’s instructions) at 4 °C overnight, followed by incubation with HRP-tagged secondary antibodies for 2 h at RT. Chemiluminescent signals were developed with Clarity ECL substrate (#170-5060; Bio-Rad) and detected using an iBright™ CL1500 Imaging System (Thermo). Blot intensities were quantified using ImageJ (NIH) software.

### ECAR and OCR estimation

The extracellular acidification rate (ECAR) and oxygen consumption rate (OCR) were analyzed as previously described (Menk et al., 2018), with minor modifications. In brief, cells were analyzed with a Seahorse XFe96 analyzer (Seahorse Bioscience) using Seahorse Wave Desktop Software (Agilent) following the manufacturer’s instructions. ECAR and OCR were measured in unstimulated cells for 1 h. Naïve CD8^+^ T cells were isolated from splenocytes from both young and old mice, counted, and 2 × 10^5^ viable cells were seeded in a 96 well seahorse assay plate coated with 2% gelatin. Cells were then stimulated by injecting media containing Dynabead-bound anti-CD3 antibody (3 µg/mL) and anti-CD28 antibody (2 µg/mL), and measurements continued for an additional 4 h.

### Analysis of PDH phosphorylation

Phosphorylation of PDH E1α at Ser232 was quantified using an ELISA kit (ab115343, Abcam) according to the manufacturer’s instructions. Briefly, protein lysates of naïve CD8^+^ T cells isolated from young and aged mice were prepared using an extraction buffer supplemented with protease inhibitors, and protein concentration was measured prior to analysis. Equal amounts of protein were loaded into pre-coated 96-well plates and incubated to allow PDHA1 binding. After washing, a primary antibody recognizing PDHA1 phosphorylated at Ser232 was added, followed by incubation with HRP-conjugated secondary antibody. Signal was developed using a chromogenic substrate and measured at 450 nm using a microplate reader. Phospho-PDHA1 levels were normalized to total protein input.

### RNA and qPCR

Total RNA was purified from cells using ISOGEN (#311-02501, Nippon Gene) reagent. cDNA was synthesized using a ReverTra Ace qPCR Kit (#FSQ-101, Toyobo), and the qPCR was performed with KAPA SYBR fast qPCR kit master mix (#KK4602, Kapa Biosystems) and a StepOnePlus RT-PCR (Applied Biosystems) system. Details of all primers used are listed in **Supplementary Table S3**.

### CRISPR/Cas9 nucleofection assay

The Cas9 ribonucleoprotein (RNP) complex was prepared as described previously (35). Briefly, 1 nmol of Alt-R crRNA targeting *PDHK1* (crPDHK1-1, GGCGGGGCTCGGTATGAGGC, and crPDHK1-2, GGCTCGGTATGAGGCTGGCA; IDT) or negative control Alt-R crRNA (crNTC, 1072544; IDT) was mixed separately with 1 nmol of Alt-R tracrRNA (1072535; IDT) for 10 min at RT. RNA mix was then annealed at 95 °C for 5 min using a thermocycler and allowed to cool to 25 °C. To prepare RNP, 150 pmol crRNA:tracRNA duplex was mixed with 60 pmol Cas9 protein (A36498; Invitrogen) for 10 min at RT. Naïve CD4^+^ or CD8^+^ T cells were nucleofected with control RNP or PDHK-targeting RNP using a P4 primary cell nucleofector kit (V4XP-4024; Lonza) according to the procedure described earlier (35). About 1x10^7^ naïve T cells were washed with PBS, added with 20 μL of P4 nucleofector solution, and mixed with 5 μL of RNP complex at RT. The cell and RNP mixture were then loaded into nucleofection cuvette, and electroporated using a Lonza 4D Nucleofector X unit (program: DS137). Cells were then transferred into 96-well plates by adding 200 μL of pre-warmed medium to each cuvette well. Cells were then maintained in complete medium supplemented with 5 ng/mL of IL-7 (577802; Biolegend) for 48 h before their activation/polarization.

### Retrovirus infection

To overexpress mouse PDHK1, C-terminally Flag-tagged PDHK1 (gene ID: 228026) was synthesized (IDT) and cloned into the retroviral MIGR1 plasmid (Addgene; #27490), generating MIGR1-PDHK1, which bicistronically express PDHK1 and a GFP reporter. The retrovirus production and transduction to activated mouse T cells were performed as described previously (36). In brief, 5 μg of MIGR1-PDHK1 or MIGR1 empty vector together with pCL-Eco (Addgene, # 12371) in 250 μL Opti-MEM were mixed with 25 μL of polyethylenimine (1 mg/mL; Cosmobio) in 250 μL of Opti-MEM (DNA: PEI-1:5 ratio), incubated at RT for 30 min, and then added to the Plat-E cells. After 24 h of transfection, the medium was replaced with fresh medium. At 72 h post-transfection, the supernatant was collected, filtered, and centrifuged at 24,000 x g for 2 h at 4 °C. The virus pellet was resuspended in medium. 24-well plates coarted with retronectin (Takara Bio, Japan) overnight at 4 °C was blocked with 2% BSA for 30 min at RT. Concentrated virus was resuspended in 400 μL medium and transferred to wells of the plate and centrifuged at 2,000 x g for 2 h at 32 °C. Cells preactivated with anti-CD3 and anti-CD28 antibodies were added into wells containing Retronectin-bound virus, and the plate was centrifuged at 300 x g for 10 min at 32 °C and incubated for 48 h. Infected cells were collected and resuspended into fresh medium containing at 0.5 x10^6^ cells/mL dilution. Cells were cultured for 6–8 days with medium replaced every other day. GFP^+^ cells were sorted using FACS and used in subsequent assays.

### Statistical analysis

Statistical significance was determined using diverse methods as indicated in each figure legend. For quantitative proteomic analysis, p values and FDR were calculated using a two-tailed unequal variance (Welch’s) t-test, and p-values were transformed to −log10 scale. Proteins with a fold change >2 and *p < 0.05* were considered significant. For GO and pathway enrichment analyses, −log10(FDR) values representing significant alterations were computed using the ShinyGO tool (v0.85). Expression differences between control and test groups in mass spectrometry, immunoblotting, qPCR, and flow cytometry data were assessed using a two-tailed unpaired t-test or one-way ANOVA with Tukey’s *post hoc* test in GraphPad Prism (version 9). P values < 0.05 were considered statistically significant and are denoted as *p < 0.05, **p < 0.01, and *p < 0.001.

## Data Availability

The MS proteomics data files have been deposited to the Japan Proteome Standard Database (jPOST) repository (37), a partner of ProteomeXchange Consortium. The accession numbers of the MS data are JPST004325 https://repository.jpostdb.org/entry/JPST004325.7 for jPOST and PXD073338 http://proteomecentral.proteomexchange.org/cgi/GetDataset?ID=PXD073338 for ProteomeXchange.

## Supplementary material

Supplementary Figure S1 DIA-MS revealed proteomic profiles across young and aged naïve and activated CD4+ T cells. **(A)** Extracted naïve and activated protein fractions from young and aged CD4^+^ T cells as resolved on SDS-PAGE and visualized with Coomassie blue staining. **(B)** Total ion chromatogram (TIC) overlay plot for each acquired sample in triplicate. **(C)** Graph showing precursor coefficient of variations (CVs) below X; therein precursor counts for identified and identified with varied CVs across the 4 samples/conditions. **(D)** Heatmap showing identified protein groups (PGs) IDs across all 4 triplicated samples/runs. **(E)** Graph showing precursor profiles with 98.9% recovery, reflected in the number of identifications. **(F)** Normalized response values as analyzed by the Spectronaut. **(G)** Protein FDR and p-value (inset) for identified proteins (verified identifications). Proteins were identified with 1% FDR (or 0.01 q-value) using Target-Decoy Competition (TDC) method) **(H)** Graph shows ranked identified proteins (candidates and PGs) based on their normalized quantity. **(A–H)** The plots represent data from n = 3 biological replicates/mice, each for young and aged naïve or activated CD4^+^ T cells.

Supplementary Figure S2 Expression of significantly altered proteins in naïve and activated subsets of young and aged CD4^+^ T cells. **(A)** Plotted (heatmap) normalized quantities of significantly altered proteins in aged and young naïve CD4^+^ T cells. **(B)** Plotted normalized quantities of significantly altered proteins in aged and young activated CD4^+^ T cells. **(A, B)** The plots represent data from n = 3 biological replicates/mice, each for young and aged naïve **(A)** or activated **(B)** CD4^+^ T cells.

Supplementary Figure S3 Glycolysis and glutaminolysis cellular processes in aged CD4^+^ T cells. **(A, B)** Plotted normalized quantities of candidate proteins altered in glycolysis **(A)** and glutaminolysis **(B)** pathways in the naïve and activated subsets of young and aged CD4^+^ T cells. **(A, B)** The plots represent data from n = 3 biological replicates/mice, each for young and aged naïve or activated CD4^+^ T cells.

Supplementary Figure S4 Immunoblots showing PDHK1 expression in young and aged activated Th1, Th2, Th17, and naïve CD8^+^ T cells. **(A, B)**. PDHK1 and β-actin full blots in young and aged activated Th1, Th2, Th17 **(A)** and naïve CD8+ T cells **(B)**.

Supplementary Figure S5 PDHK1 inhibition impacts T cell activation and viability, but not cell proliferation. **(A)** Flow cytometry analysis of CD25 and CD69 expressions in naïve and 12 h activated control (untreated) and DCA-treated young CD4^+^ T cells. **(B)** Flow cytometry-based cell viability analysis in Con-KO and PDHK1-KO activated CD4^+^ T cells at 48h timepoints as detected by zombie staining. **(C)** Flow cytometry analysis of CFSC-based cell proliferation in Con, Con-KO, and PDHK1-KO CD4^+^ T cells at 0 h, 24 h, 48 h, 72 h, and 96 h timepoints. **(A, B)** The plots represent data from n = 3 biological replicates, each for young naïve and activated **(A)** or Con, Con-KO, and PDHK1-KO CD4^+^ T cells.

Supplementary Figure S6 PDHK1 overexpression improves activation, survival, and effector molecule expression in aged T cells. **(A)** Flow cytometry dot plots of CD25 and CD69 expression in T helper cells transduced with Flag-PDHK1. CD4^+^ T cells transduced with retroviral vectors were cultured for 48 h after infection, followed by polarization to T helper subsets for 48 h prior to FACS analysis. **(B)** Flow cytometry histograms of CD44 expression in T helper and CD8^+^ T cells transduced with Flag-PDHK1. Cells were transduced and activated as described in A. C-E. Flow cytometry histogram plots of IFN-γ expression **(C)**, TNFα, Granzyme B, and Perforin-1 expression **(D)**, frequency of zombie+ dead cells (E, contour plot), in CD8^+^ T cells transduced with Flag-PDHK1. CD4^+^ T cells transduced with retroviral vectors were cultured for 48 h after stimulation with anti-CD3 and anti-CD28 antibodies. 48 h later, cells were restimulated with PMA/ionomycin for 4 h, and IFN-γ TNFα, Granzyme B, and Perforin-1 expressions were analyzed **(C, D)**.

Supplementary Table S1 Details of the total identified 3504 unique proteins across the 4 cell types.

Supplementary Table S2 Details of the quantitative altered proteins across the 4 cell types.

Supplementary Table S3 List of qPCR primers used in this study.
