# Correction: Age-related reduction of pyruvate dehydrogenase kinase 1 impairs T cell responses

**DOI:** 10.3389/fimmu.2026.1892200

**Published:** 2026-06-04

**Authors:** Rajkumar S. Kalra, Miho Tamai, Shukla Sarkar, Yong-Woon Han, Mio Miyagi, Daiki Sasaki, Hiroki Ishikawa

**Affiliations:** 1 Immune Signal Unit, Okinawa Institute of Science and Technology, Graduate University (OIST), Onna-son, Okinawa, Japan; 2 Department of Molecular Biosciences, Radiation Effects Research Foundation, Hiroshima, Japan; 3 Department of Biological Science, Faculty of Science and Engineering, Yasuda Women’s University, Hiroshima, Japan; 4 Laboratory for Integrative Genomics, RIKEN Center for Integrative Medical Sciences (IMS), Yokohama, Kanagawa, Japan; 5 Department of Molecular Oncology, Nagoya City University Graduate School of Medical Sciences, Nagoya, Japan

**Keywords:** aerobic glycolysis, aging, DIA-MS, metabolism, PDHK1, T cell activation

Affiliation “Department of Molecular Biosciences, Radiation Effects Research Foundation, Hiroshima, Japan” was omitted for author Rajkumar S. Kalra and instead was added solely as a present address. This affiliation has now been added to the affiliations list and removed from the present address section.

The affiliation “Department of Biological Science, Faculty of Science and Engineering, Yasuda Women’s University, Hiroshima, Japan” was erroneously added to Rajkumar S. Kalra. It has now been removed.

In the **Acknowledgements**, a statement regarding the current employment of author Rajkumar S. Kalra was omitted. The **Acknowledgments** has been updated to read:

“We thank the personnel of the Instrumental Analysis Section (IAS), especially of Alejandro Villar-Briones, Dr. Yoshitoshi Hirao, and Dr. Michael C. Roy, for their support. Also, we thank the staff of the Animal Resources Section (ARS) at Okinawa Institute of Science and Technology Graduate University for their help in animal-related support. RSK acknowledges the support from RERF. This work was performed without financial support from the RERF, and the views expressed do not necessarily reflect those of the institution.”

The original version of this article has been updated.

