# Supplementary material for: Age-related reduction of pyruvate dehydrogenase kinase 1 impairs T cell responses
Source: Front Immunol. 2026 May 11;17:1800870. doi: 10.3389/fimmu.2026.1800870 (PMC13200556; doi:10.3389/fimmu.2026.1800870)
Supplement: Supplementary Figure S1 — DIA-MS revealed proteomic profiles across young and aged naïve and activated CD4+ T cells. (A) Extracted naïve and activated protein fractions from young and aged CD4+ T cells as resolved on SDS-PAGE and visualized with Coomassie blue staining. (B) Total ion chromatogram (TIC) overlay plot for each acquired sample in triplicate. (C) Graph showing precursor coefficient of variations (CVs) below X; therein precursor counts for identified and identified with varied CVs across the 4 samples/conditions. (D) Heatmap showing identified protein groups (PGs) IDs across all 4 triplicated samples/runs. (E) Graph showing precursor profiles with 98.9% recovery, reflected in the number of identifications. (F) Normalized response values as analyzed by the Spectronaut. (G) Protein FDR and p-value (inset) for identified proteins (verified identifications). Proteins were identified with 1% FDR (or 0.01 q-value) using Target-Decoy Competition (TDC) method) (H) Graph shows ranked identified proteins (candidates and PGs) based on their normalized quantity. (A–H) The plots represent data from n = 3 biological replicates/mice, each for young and aged naïve or activated CD4+ T cells. [file DataSheet1.pdf]

Supplementary Material

Age-related reduction of pyruvate dehydrogenase kinase 1 (PDHK1) impairs T cell responses

Rajkumar S. Kalra[1,2†*], Miho Tamai[1,3], Shukla Sarkar[1], Yong-Woon Han[4,5], Mio Miyagi[1], Daiki Sasaki[1], and Hiroki Ishikawa[1,3*]

Supplementary Figures legends-

Figure S1. DIA-MS revealed proteomic profiles across young and aged naïve and activated CD4+ T cells.

A. Extracted naïve and activated protein fractions from young and aged $CD4^+$ T cells as resolved on SDS-PAGE and visualized with Coomassie blue staining.

B. Total ion chromatogram (TIC) overlay plot for each acquired sample in triplicate.

C. Graph showing precursor coefficient of variations (CVs) below X; therein precursor counts for identified and identified with varied CVs across the 4 samples/conditions.

D. Heatmap showing identified protein groups (PGs) IDs across all 4 triplicated samples/runs.

E. Graph showing precursor profiles with 98.9 % recovery, reflected in the number of identifications.

F. Normalized response values as analyzed by the Spectronaut.

G. Protein FDR and p-value (inset) for identified proteins (verified identifications). Proteins were identified with 1% FDR (or 0.01 q-value) using the Target-Decoy Competition (TDC) method)

H. Graph shows ranked identified proteins (candidates and PGs) based on their normalized quantity.

(**A-H**) The plots represent data from n = 3 biological replicates/mice, each for young and aged naïve or activated $CD4^+$ T cells.

Figure S2. Expression of significantly altered proteins in naïve and activated subsets of young and aged $CD4^+$ T cells.

A. Plotted (heatmap) normalized quantities of significantly altered proteins in aged and young naïve $CD4^+$ T cells.

B. Plotted normalized quantities of significantly altered proteins in aged and young activated $CD4^+$ T cells.

(**A-B**) The plots represent data from n = 3 biological replicates/mice, each for young and aged naïve (A) or activated (B) $CD4^+$ T cells.

Figure S3. Glycolysis and glutaminolysis cellular processes in aged $CD4^+$ T cells.

A-B. Plotted normalized quantities of candidate proteins altered in glycolysis (A) and glutaminolysis (B) pathways in the naïve and activated subsets of young and aged $CD4^+$ T cells.

(**A-B**) The plots represent data from n = 3 biological replicates/mice, each for young and aged naïve or activated $CD4^+$ T cells.

Figure S4. Immunoblots showing PDHK1 expression in young and aged activated Th1, Th2, Th17, and naïve $CD8^+$ T cells.

A-B. PDHK1 and β-actin full blots in young and aged activated Th1, Th2, Th17 (A) and naïve CD8+ T cells (B).

Figure S5. PDHK1 inhibition impacts T cell activation and viability, but not cell proliferation.

A. Flow cytometry analysis of CD25 and CD69 expressions in naïve and 12 h activated control (untreated) and DCA-treated young $CD4^+$ T cells.

B. Flow cytometry-based cell viability analysis in Con-KO and PDHK1-KO activated $CD4^+$ T cells at 48h timepoints as detected by zombie staining.

C. Flow cytometry analysis of CFSC-based cell proliferation in Con, Con-KO, and PDHK1-KO $CD4^+$ T cells at 0 h, 24 h, 48 h, 72 h, and 96 h timepoints.

(**A-B**) The plots represent data from n = 3 biological replicates, each for young naïve and activated (A) or Con, Con-KO, and PDHK1-KO $CD4^+$ T cells.

Figure S6. PDHK1 overexpression improves activation, survival, and effector molecule expression in aged T cells.

Supplementary Material

A. Flow cytometry dot plots of CD25 and CD69 expression in T helper cells transduced with Flag-PDHK1. $CD4^+$ T cells transduced with retroviral vectors were cultured for 48 h after infection, followed by polarization to T helper subsets for 48 h prior to FACS analysis.

B. Flow cytometry histograms of CD44 expression in T helper and $CD8^+$ T cells transduced with Flag-PDHK1. Cells were transduced and activated as described in A.

C-E. Flow cytometry histogram plots of IFN-γ expression (C), TNFα, Granzyme B, and Perforin-1 expression (D), frequency of zombie+ dead cells (E, contour plot), in $CD8^+$ T cells transduced with Flag-PDHK1. $CD4^+$ T cells transduced with retroviral vectors were cultured for 48 h after stimulation with anti-CD3 and anti-CD28 antibodies. 48 h later, cells were restimulated with PMA/ionomycin for 4 h, and IFN-γ TNFα, Granzyme B, and Perforin-1 expressions were analyzed (C-D).

Supplementary Tables legends-

Table S1. Details of the total identified 3504 unique proteins across the 4 cell types.

Table S2. Details of the quantitative altered proteins across the 4 cell types.

Table S3. List of qPCR primers used in this study.

Supp Figure 1

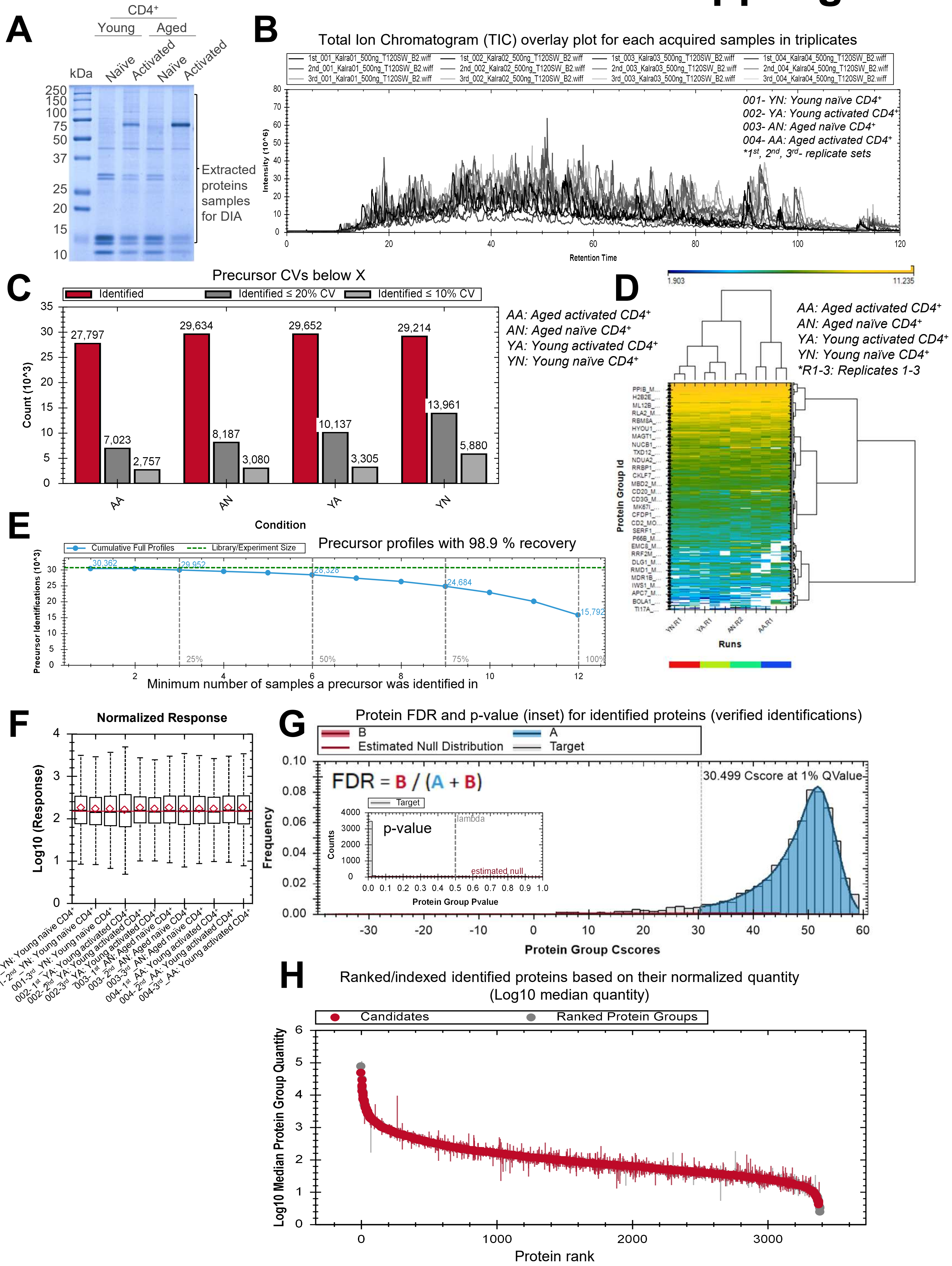

Supp Figure 2

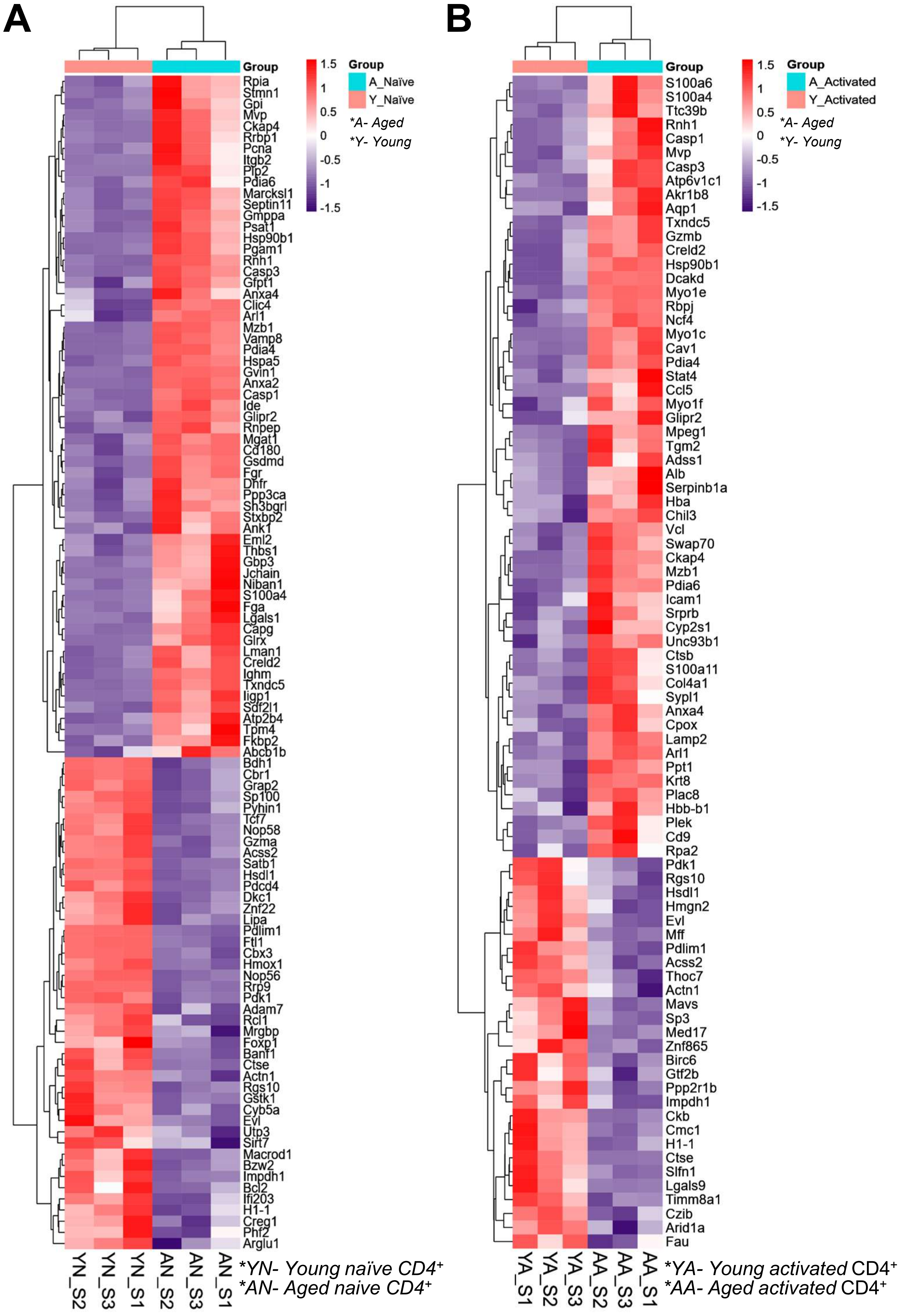

Supp Figure 3

A

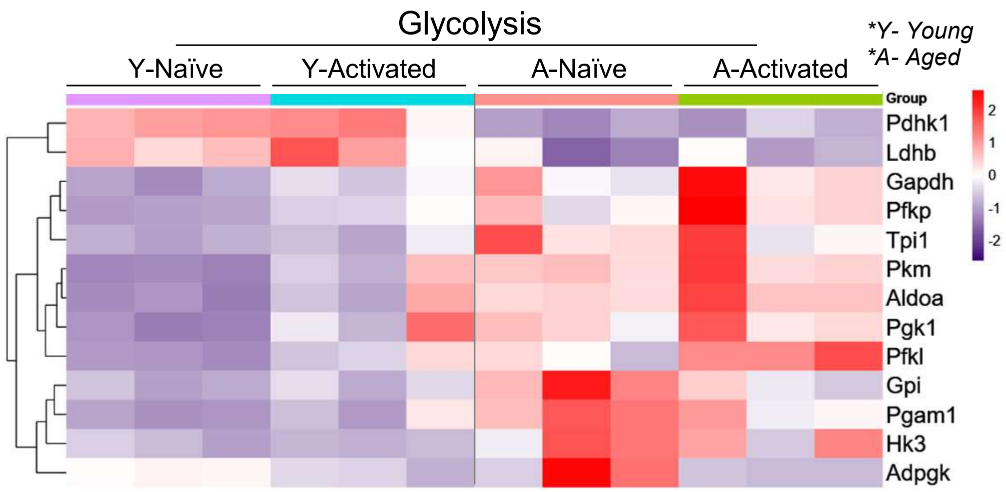

B

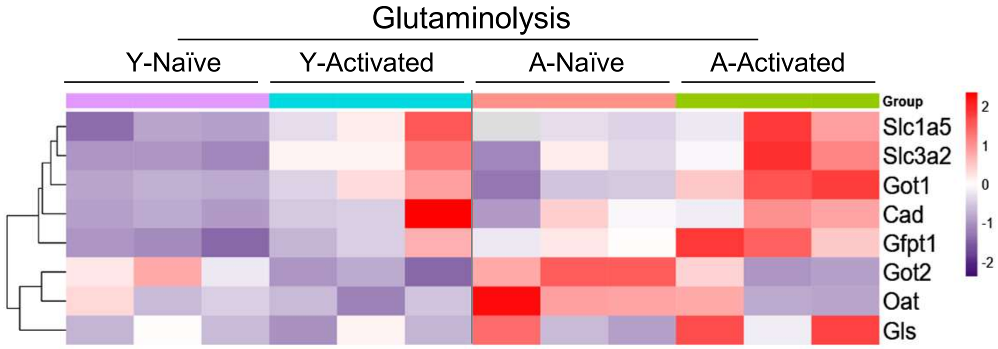

Supp Figure 4

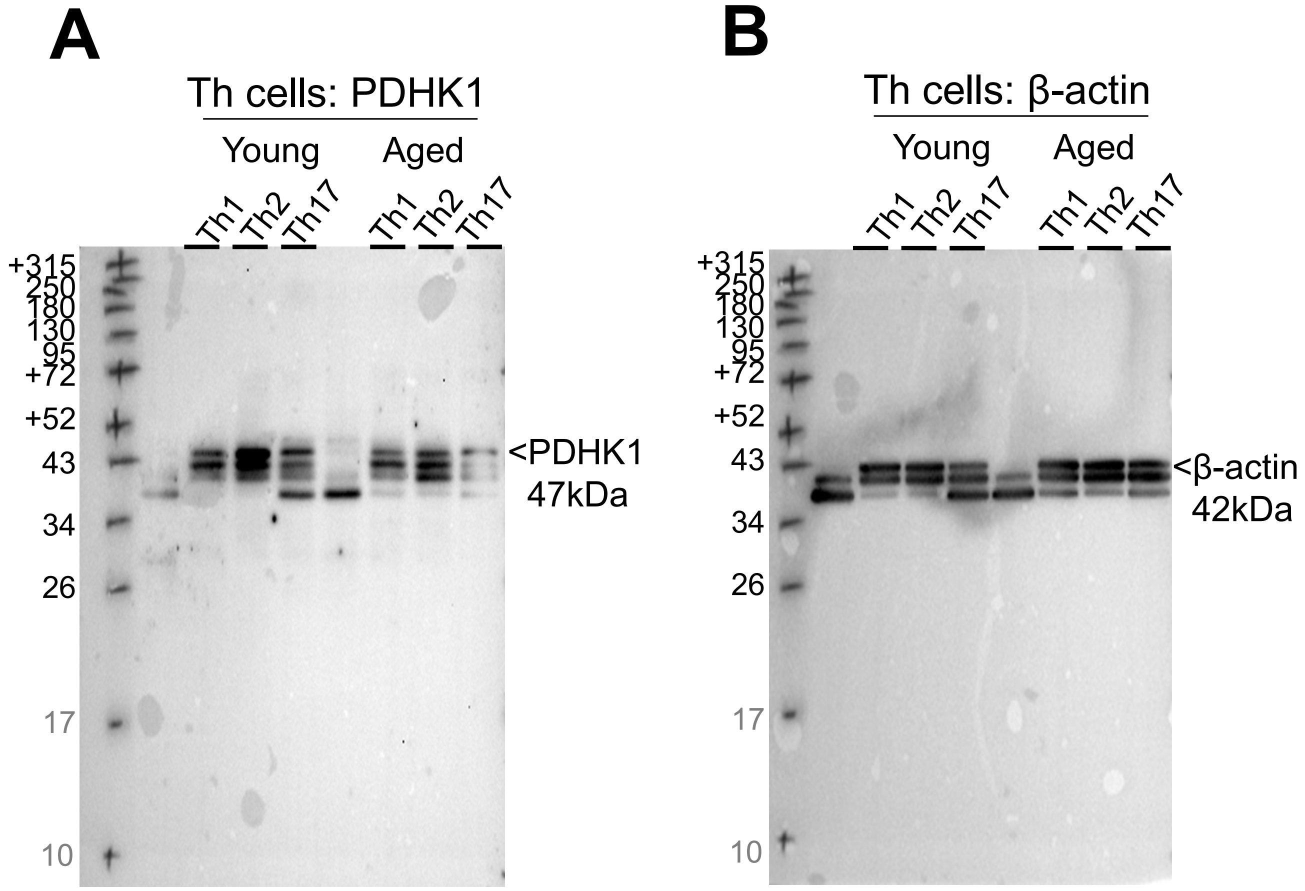

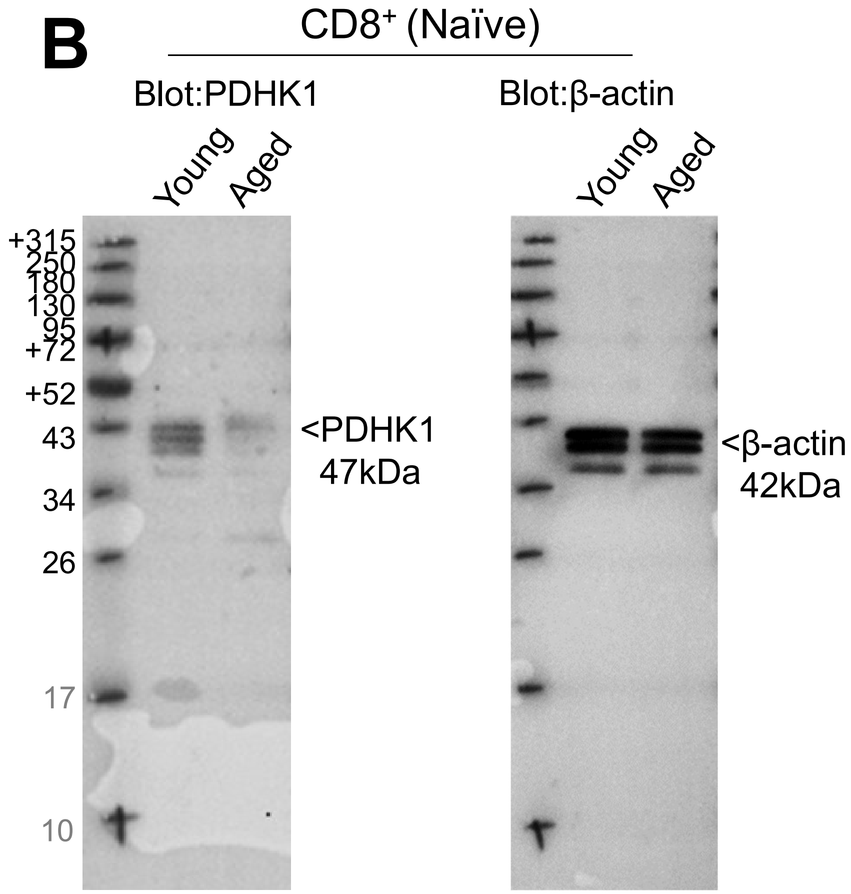

Supp Figure 5

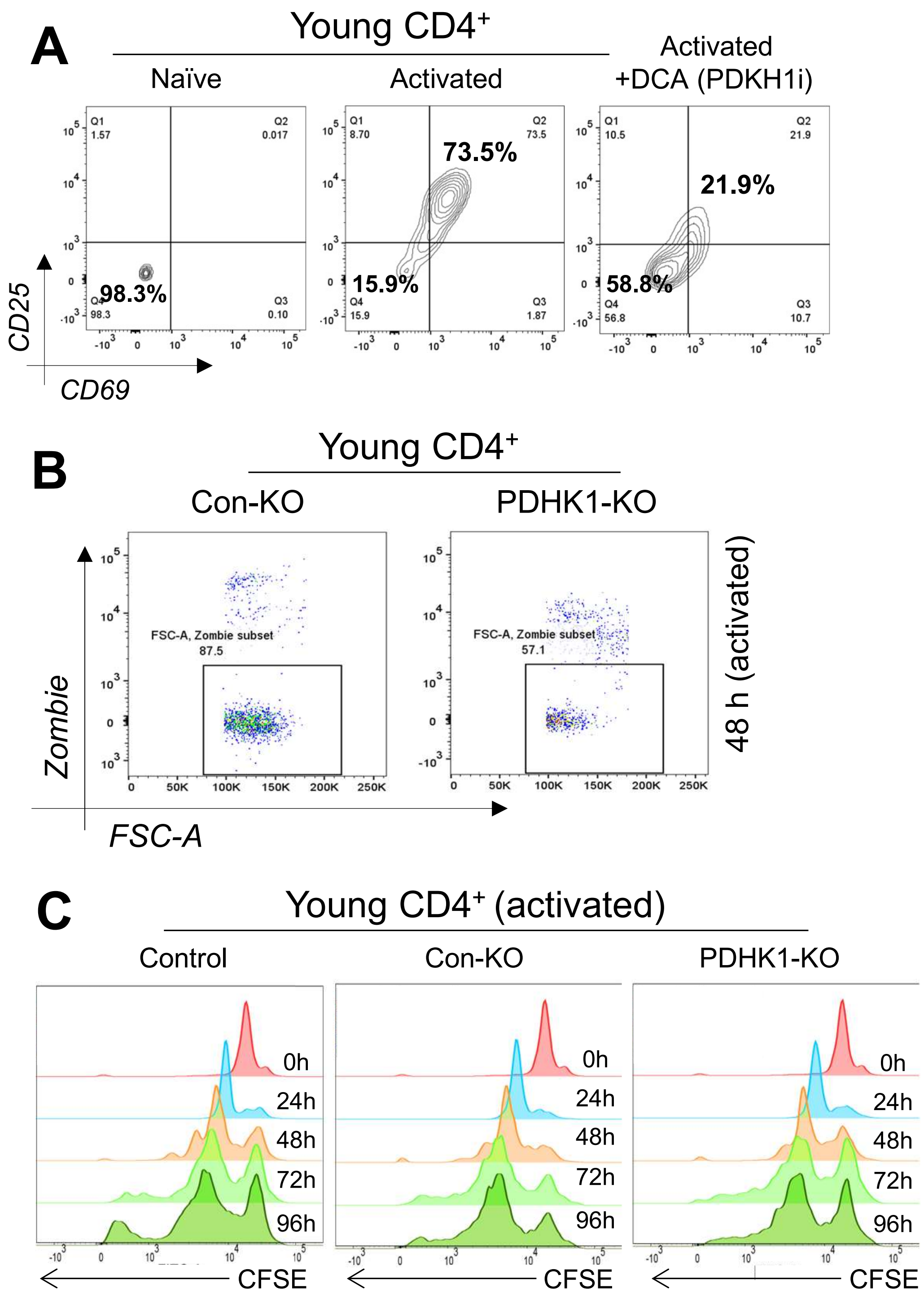

Supp Figure 6

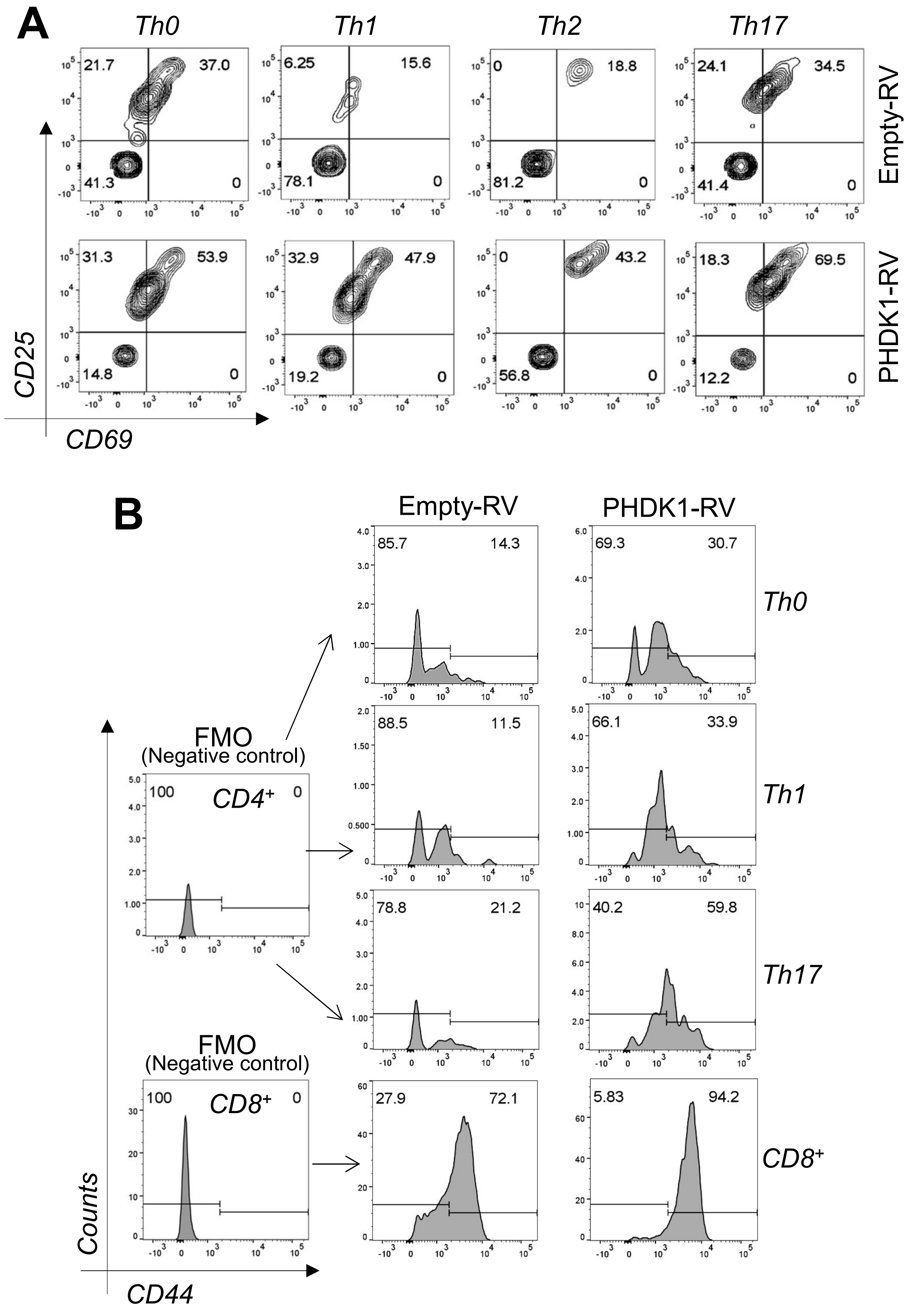

Supp Figure 6

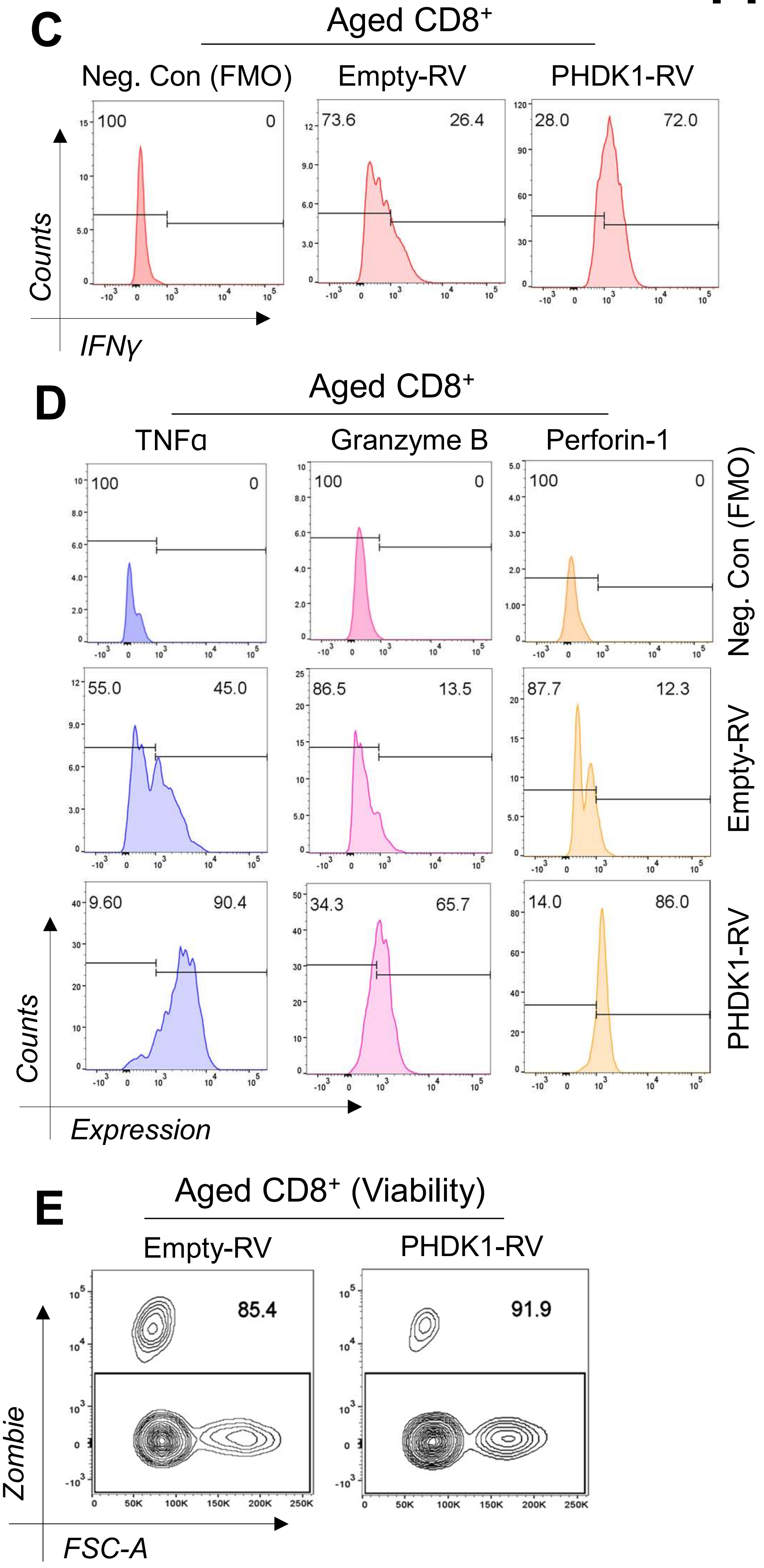

Table S3. List of qPCR primers used in this study.

	Gene	Sequence
qPCR	Ms-PDK1-F	TCCCCCGATTCAGGTTCAC
	Ms-PDK1-R	CCCGGTCACTCATCTTCACA
	Ms-PDK2-F	TGGACCGCTTCTACCTCAG
	Ms-PDK2-R	TCTTTCACCACATCAGACACG
	Ms-PDK3-F	GCCCAAGGCGTGATTGAGTA
	Ms-PDK3-R	GGGTTAGTGTCACCACCAAAC
	Ms-PDK4-F	ATCTAACATCGCCAGAATTAAACC
	Ms-PDK4-R	GGAACGTACACAATGTGGATTG
	Ms-HPRT-F	GAAGAGCTACTGTAATGATCAGTCAAC
	Ms-HPRT-R	AGCAAGCTTGCAACCTTAACCA
